# The Effect of Experience on Anxiety in Food Safety Incidents—An Empirical Study on Infant Formula Safety Incidents in China

**DOI:** 10.3390/healthcare10010138

**Published:** 2022-01-12

**Authors:** Ke LI, Xueyan Cao, Zhiwei He, Liqun Liu

**Affiliations:** 1 School of Journalism and Communication, Wuhan University, Wuhan 430072, China; worbych@whu.edu.cn (K.L.); caoxueyanluojia@whu.edu.cn (X.C.); hezhiwei@yandex.com (Z.H.); 2 Center for Studies of Media Development, Wuhan University, Wuhan 430072, China; 3 National Institute of Cultural Development, Wuhan University, Wuhan 430072, China

**Keywords:** infant formula, risk experience, anxiety, risk perception, perceived knowledge gap

## Abstract

Infant formula incidents have endangered the dietary safety and healthy growth of infants and young children and are triggers of the public’s negative emotions, attracting widespread public attention. The aim of this research was to explore how perceived knowledge gap, risk perception, past actual risk experience, and media risk experience affect anxiety. The research data obtained from 506 respondents were divided into groups with actual risk experience and without actual risk experience. Then, PLS-SEM was used to analyze the data. The results show that risk perception mediated the relationship between perceived knowledge gap and anxiety. Specifically, for the group with actual risk experience, perceived knowledge gap had a significant direct impact on anxiety; however, there was no moderation effect of media experience on the relationship between perceived knowledge gap and risk perception. For the group without actual risk experience, perceived knowledge gap had no direct effect on anxiety, and media experience had a significant moderating effect on the relationship between perceived knowledge gap and risk perception. The results suggest that in infant formula safety incidents, actual risk experience and media risk experience have different influence mechanisms on anxiety. Actual risk experience will directly and intuitively bridge the relationship between perceived knowledge gap and anxiety. Meanwhile, groups without actual risk experience tend to be influenced by rational risk judgment, and this process is moderated by media risk experience.

## 1. Introduction

Food safety incidents, as one of the common challenges facing the world, pose a threat to personal life and global public health. Among many food safety incidents, those involving infant food have attracted special attention from society. Society is especially concerned with safety incidents related to infant formula. First of all, the food choice of infants and young children is very narrow. Infant formula plays a vital role in the dietary source, and it is the main, or even the only, nutritional source for many infants and young children. Secondly, problematic infant formula may cause long-term and catastrophic consequences for infants and young children. However, due to children’s inability to express themselves, risks are often discovered after severe consequences have been caused. Third, the decision making of infants’ and young children’s dietary choices is mainly made by parents. Infants and young children themselves cannot provide sufficient feedback. Therefore, parents can only make dietary choices based on experience or relevant knowledge. This decision is quite difficult for some parents who lack relevant knowledge. Fourth, due to the high sensitivity of the public to food safety incidents involving infants and young children, once the incidents occur, significant social repercussions generally appear in a short time, causing strong negative group emotions among the public. As a result, the consumption decision of infant formula, parents’ infant feeding decision, and even the trust in relevant policies and regulations will be affected.

In China, the safety of infant formula is a particular concern of the government and the public. The reason is that there has been more than one serious safety incident of infant formula in the past two decades, attracting extensive social attention every time. In 2003, many babies suffered from “big head disease” in Fuyang, Anhui Province. The infants’ heads swelled while their bodies did not grow properly. It was reported that these infants were fed fake formula without nutritional value, resulting in 190 malnourished infants. A total of 13 infants died, and 6 officials were sacked over the safety incident [1]. In 2008, Sanlu infant formula adulterated with the chemical raw material melamine caused infants to suffer from kidney stones and other diseases. It caused the deaths of 6 babies, and there were approximately 294,000 victims; the incident resulted in two executions, and three sentences of life imprisonment [2]. Since then, the public’s risk perception of domestic dairy products has increased sharply, and consumption has remained depressed; therefore, these incidents have triggered huge changes in Chinese food safety systems. From 2015 to 2019, there were continuous reports of fake baby formula of well-known overseas brands; one of the relevant news items was that Spain shut down a factory packaging fake infant formula originally destined for China in 2018 [3]. This incident caused great worry among Chinese parents who are willing to pay a premium for imported overseas formula. From the above infant formula safety incidents, it can be seen that infant formula safety issues affect consumers’ consumption choices. The repeated incidents concerning domestic infant formula in the early stages led to lower consumer confidence, and as a result of the damaged reputation, Chinese consumers turned to imported overseas infant formula. However, this surge in the consumption of imported infant formula enabled businesses to make huge profits by counterfeiting imported infant formula. On the other hand, it was purely human error to blame for the three incidents. Moreover, the repeated incidents increased the public’s negative emotions towards risk-related responsible parties [4]. Gong and Jackson investigated the anxiety of parents after the Sanlu infant formula safety incident in 2008 and found that the incident affected parents’ parenting practice (turning to expensive imported infant formula, relying on a supply of dairy products from relatives and friends, etc.) [5]. Consequently, anxiety about infant formula safety incidents persists, which has a profound impact on parents’ infant feeding decisions. Therefore, from the social perspective, the harm of group anxiety caused by the safety of infant formula is even more serious than the direct harm. Reducing excessive group anxiety in food safety events as much as possible has become a critical issue.

As the relevant departments of the Chinese government will act immediately when a food safety issue occurs, it is rare for a certain type of food to have repeated serious safety problems, which indicates that the public’s knowledge and risk experience of specific food safety issues will not accumulate. However, repeated occurrences and significant impacts have accumulated in the public’s relevant knowledge, experience, and emotion regarding the infant formula issue. Individuals who were not affected by the tainted infant formula have also formed a specific risk experience through various media messages. This objectively makes it an important research case related to food safety risk experience. This study aimed to analyze the influencing factors of public anxiety and the role of risk experience of infant formula safety incidents to explore the mechanism of negative social emotions represented by anxiety under food safety incidents.

## 2. Literature Review

### 2.1. Anxiety

Anxiety is a subjective feeling of uneasiness, worry, or fear, accompanied by many physical manifestations. It is a normal, emotional, reasonable, and predictable response to real or potential threats. It arises from uncertainty assessment and the need to control the current situation [6,7]. Negative emotions represented by anxiety are generally aroused by external risks and change with the development of specific risk events. Anxiety is regarded as one of the important variables in the related research of risk communication. Meanwhile, anxiety is associated with negative risk assessment, which affects the tendency to engage in risk-avoidant decision making [8]. Accordingly, negative judgments about the future lead to risk aversion [9]. In terms of food safety risks, Li et al. showed that negative emotions caused by food safety incidents had a negative impact on consumers’ food purchase intention [10]. Jin et al. further demonstrated that negative emotions may urge consumers to change their food purchase strategies and turn to other alternatives [11]. Additionally, Frijda believed that motivational emotions, especially negative emotions such as anxiety, can cause individuals to generate information behavior tendencies, make behavior preparations, and finally regain control of the situation by accepting and searching for risk information that leads to negative emotions [12]; this has also been confirmed by many subsequent studies [13,14,15]. Therefore, it is plausible that public anxiety and other negative emotions can easily lead to major public concern over food safety issues.

Regarding the cause of anxiety, both cognitive theory and evaluation theory attribute it to the subjective evaluation of the individual, which is generally understood as an emotion triggered by the uncertainty of the current situation and the development of the events [16]. Turner et al. developed an anxiety reduction hypothesis based on the risk perception attitude framework. They believed that negative emotions such as fear and anxiety stemmed from situations in which an individual perceived the severity of the risk and their own weak resistance [17]. Research confirms that people with higher risk perception have higher levels of anxiety. Hanna et al. found that overestimation of the risk of food allergies by health experts plays a role in health transmission and can lead to increased anxiety among parents [18]. These research results suggest that anxiety in food safety risk events mainly comes from the influence of individuals’ perception and subjective assessment of risks.

### 2.2. Perceived Knowledge Gap

Food safety incidents urge consumers to acquire knowledge of food quality, safety, and nutritional content. As the key factor of food risk communication, knowledge includes objective knowledge and subjective knowledge, which may have a direct or indirect influence on consumers’ food cognition, attitude, and behavioral decision-making process. In the prediction of consumer behavior, consumers’ subjective knowledge plays a more important role than objective knowledge [19]. Notably, Luten emphasized that subjective knowledge had a better predictive effect on fish consumption as compared with objective knowledge [20]. In Poland and Spain, where consumers have the highest subjective knowledge, it was found that there is no significant correlation between objective knowledge and fish consumption level. Subjective knowledge has been proved to matter as a determinant in functional food consumption [21,22].

In risk events, anomalous state of knowledge (ASK) theory assumes that individuals’ recognition of ambiguous and ill-defined descriptions of the need for information in a problematic situation is the source of information demand and the change in information behavior [23]. Dervin internalized this abnormal state of knowledge as a gap [24]. In the information search and processing model, Griffin introduced the concept of information sufficiency to illustrate the gap between the current knowledge level felt by the individual and the knowledge level the individual felt as sufficient to respond to the risk adequately [25]. This “knowledge gap” stems from the individual’s cognitive evaluation of the current environment. It is a lack of subjective knowledge, rather than a lack of objective knowledge based on professional evaluation. According to evaluation theory, the individual is uneasy about the current state of knowledge, that is, when the perceived knowledge is insufficient, it may directly lead to the generation of negative emotions [26]. Nabi RL confirmed that the lower the level of perceptual knowledge, the higher the arousal of negative emotions [27]. Lee et al. showed that insufficient perceived knowledge of patients and their families about cancer risk can lead to increased anxiety [28].

In infant formula safety incidents, the primary victims are infants and young children who have not yet formed a clear self-perception and mature decision-making ability. At this stage, the responsibility for risk decision making is mainly borne by their parents and other relatives. Relevant knowledge is the main, or even the only, basis for parents’ feeding decision making, while parents’ subjective evaluation of their knowledge level will affect the emotional response to food safety issues. For the non-victims, it is also easy to identify a strong emotional response because the victims are infants and young children. Compared with the parents of infants and young children, it is plausible that these non-victims’ perceived knowledge gap and even their relevant knowledge may be scarcer, which may lead to anxiety. Thus, the following hypothesis was derived:

**Hypothesis** **1** **(H1).** *The perceived knowledge gap in the risks of infant formula has a significant positive impact on anxiety*.

### 2.3. Risk Perception

Risk perception is a cognitive response triggered by an external situation. It involves subjective judgments on two different dimensions: possibility and severity. The former dimension refers to the possibility that a person will encounter similar problems, while the latter dimension refers to the severity of consequences of similar problems [29]. Sitkin and Pablo argued that risk perception was a highly analytical and rational process, which means that individuals make reasonable judgments about the correlation between themselves and risk events [30].

Risk perception has been discussed from the perspective of prospect theory [31], differences in individual cognitive models [32], and cultural theory [33]. Risk perception is considered as a common result driven by individual feedback on risk events and the external socio-economic environment. However, the formation of individual risk perceptions will not completely follow a single mechanism. Especially in terms of food safety issues, a series of individual subjective perceptions will have a significant impact on risk perception [34]. Schroeder et al. defined food safety risk perception (FSRP) as an individual’s sensitivity to food safety and the severity of health problems caused by its consumption [35]. A meta-analysis found that perceived knowledge was one of the key drivers of food safety risk perception and had a strong negative impact on food purchase intentions [36]. Liao C et al. believed that people generally had higher perceived risks of unfamiliar things and the environment than familiar things [37]. In food recall issues, the higher the individuals’ perception of their knowledge of food safety, the lower the risk perception generated, and vice versa. Shakeri et al. [38] and Liao et al. [37] confirmed the direct impact of insufficient perceived knowledge on risk perception. It is likely that repeated infant formula safety incidents are more likely to cause consumers to fall into the dilemma of low subjective knowledge evaluation, that is, insufficient perceived knowledge, which intensifies the possibility and severity of infant formula consumption consequences, leading to higher risk perception levels. Therefore, the second hypothesis was formulated as follows:

**Hypothesis** **2** **(H2).** *The perceived knowledge gap in infant formula risk has a significant positive impact on risk perception*.

Beck proposed that it was not the risk event but the individual’s interpretation of the risk event that triggered negative emotions (such as anxiety), to a certain extent [39]. This explains the difference in the risk perception of different individuals in the same risk situation. Initially, people are aware of the occurrence of risk events, but they may overestimate or underestimate the severity of the problem, which can subsequently lead to anxiety [40]. According to the probabilistic risk analysis (PRA) framework, the perceived personal risk is the main source of anxiety. The higher the perceived personal risk, the higher the degree of anxiety [41]. The cultural model of risk emphasizes that individuals usually have inaccurate perceptions of risk, and excessive perception of low risks can trigger negative emotions and protective information behaviors [42]. The risk information seeking and processing (RISP) model proposed by Griffin confirms that risk perception directly affects the public’s emotional response to risk, such as anxiety and anger [25]. An empirical study by Kahlor tested the assumptions in the RISP model, and the results verified that risk perception can strengthen the public’s negative emotions [13]. Hence, based on the above research, the following hypothesis was proposed:

**Hypothesis** **3** **(H3).** *Risk perception of infant formula risk has a significant positive effect on anxiety*.

Mayer’s model suggests that emotion consists of the direct experience of emotion and the meta-experience level composed of thoughts and feelings about emotion [43]. The former is the immediate state of feeling under external stimuli. In infant formula safety incidents, the individual may first perceive the lack of knowledge and directly generate negative emotions such as anxiety. Meanwhile, the latter involves the reflection of the individual’s subjective feelings under self-control experience. The process in which insufficient perceived knowledge triggers anxiety by the mediating effect of risk perception is regarded as the processing of individual rational cognition. Based on the above assumptions, we proposed the fourth hypothesis, as follows:

**Hypothesis** **4** **(H4).** *Risk perception plays a mediating role in the influence of perceived knowledge gap on anxiety*.

### 2.4. Risk Experience

#### 2.4.1. Relevant Study

Generally, the experience of a risk event strengthens the memory of the risk, thereby enhancing the individual’s risk perception. Research on risk perception shows that people not only assess risk through personal experience but also judge risk by comparing it with others’ experiences, and it demonstrates a strong self-centered tendency throughout the process [44]. According to the social amplification framework theory of risk [45], the social root of risk amplification lies in experience. Knuth and Kehl found that the frequency of risk experiences and the nature of the results had a significant impact on the public’s negative emotions; specifically, the higher the frequency of experiences, the stronger the worry about involuntary risk activities [46].

Risk experience can be categorized into direct experience and indirect experience, among which direct risk experience generally makes individuals aware of their vulnerability [47], thereby triggering risk perception and negative emotions. A study by Lara et al. found that flood experience determined the public’s perception of vulnerability when facing a flood hazard [48]. Direct experience can be further subdivided into direct physical experience or emotional experience. For example, direct physical injury and psychological fear after an earthquake and tsunami will affect risk perception [49]. Among them, direct emotional experience (such as fear) directly affects the generation of negative emotions (such as worry). Studies have also found that direct experience has a stronger impact on risk perception and behavior changes than indirect experience [50]. With the development of media technology, compared with direct risk experience, the public has experienced an increasing number of risks in the media that have not been exposed in reality. In food safety incidents, direct experiences and experiences learned through the media have also been confirmed to affect people’s perception of the risk of a particular food [51]. In the infant formula safety incidents, not only were many families directly affected by problematic infant formula, but also many families were involved (for example, when an infant formula safety incident occurred, infants and young children were fed ordinary infant formula, not the problematic infant formula). This type of actual risk experience also induces negative emotions. In this study, actual experience refers to being affected by problematic infant formula. The concentrated media coverage of these incidents enables the public to obtain media risk experience to varying degrees. The impact of this indirect experience may even be more lasting, which cannot be ignored. Therefore, this study discusses actual experience and media experience separately.

#### 2.4.2. Actual Experience

In recent decades, researchers have conducted various explorations on the relationship between individual actual risk experience and risk perception. Compared with media risk experience, the impact of actual risk experience is more direct and significant. The results of Kellens et al.’s study on flood disasters showed that people who directly experienced floods had a stronger risk perception than individuals who had not experienced floods [52]. Greening et al. found that the influence of risk experience on risk perception was mediated by cognitive inspiration, that is, risk experience can affect the judgment of future risks only by evoking the memory and imagination of risk events in the human brain [53]. Actual risk experience will enhance the individual’s ability to simulate and imagine risk results again at the psychological level; in other words, risk experience reduces the perceived distance between the individual and the risk event, and its perceived relevance is therefore enhanced [53]. Combined with dual-system theory, individuals have two different processing modes for cognitive tasks: one is described as an associative, enlightening, or intuitive mode; the other is an analytical and reflective process based on rules and deductive reasoning [54]. There are differences in the generation mechanism of negative emotions between individuals with and without actual experience. Individuals with actual experience tend towards emotional perception rather than rational analysis.

In infant formula safety incidents, compared with individuals who have no actual risk experience, those who have actual experience may be more able to imagine the severity of the consequences of the incident according to their memory, assign priority to judging their knowledge level and event risk level according to their own emotion and memory, and devote less cognitive effort to choosing an inherent spontaneous processing strategy [55]. However, people without actual experience may be more affected by media risk experience and tend to use the indirect experience as the main basis for risk perception, thereby conducting a rational assessment of risk. Therefore, the fifth research hypothesis was formulated as follows:

**Hypothesis** **5** **(H5).** *There are differences in the impact of perceived knowledge gap on anxiety among individuals with different actual risk experiences*.

#### 2.4.3. Media Experience

An individual’s media risk experience partially depends on the direct risk results of others; moreover, it is also affected by the amount of risk information that the media communicates, the degree of controversy and dramatization of the information, the symbolic connotation of the information, and personal relevance [45]. Previous research has revealed that indirect media risk experience influences the individual’s risk perception, and that indirect media risk experience is regulated by risk information and the degree of self-correlation [56]. On the one hand, media risk experience will provide individuals with risk information; on the other hand, media risk experience may improve the individual’s judgment on the possibility and severity of risk events, that is, the level of risk perception. Knuth et al. suggested that the knowledge gap will not cause the individual to worry about risks, and that only when the individual judges the potential risk to be related to themself will they pay more attention to risk events [46].

For people who have no experience of infant formula safety incidents, the process of experiencing infant formula safety incidents through the media is similar to supplementing the lack of experience in reality. Media experience that is highly related to oneself is the main basis for subjective judgment of one’s knowledge level, and it may affect the strength of the relationship between perceived knowledge gap and risk perception. However, people with actual experiences may be more inclined to perceive and act according to their memories in terms of severe consequences. The influence of media experience may be relatively weakened. Nevertheless, due to the frequent occurrence of infant formula risk events and the widespread impact of media reports, media experience will still play a role. On this basis, the following hypothesis was proposed:

**Hypothesis** **6** **(H6).** *Media risk experience plays a moderating role in the influence of perceived knowledge gap on risk perception*.

## 3. Method

### 3.1. Design

Based on a cross-sectional design, this study explored the effects of infant formula safety experience on the generation of individual anxiety in mainland China and examined the differences between groups with actual risk experience and without actual risk experience through group comparisons. This research was approved and managed by the academic ethics committee.

### 3.2. Inclusion Criteria and Exclusion Criteria

This study focused on the respondents’ attitude towards the safety of infant formula, and thus it had certain requirements for the respondents. First of all, considering that the subject of the study required the respondents to have certain social behavior abilities and judgment, subjects under 18 years old were not included in the present study. Secondly, respondents needed to have some knowledge about infant formula, or, at least, they needed to show interest in the relevant information. Although it is generally believed that parents of infants and young children are most concerned about the issue of infant formula, it has been found in a preliminary survey that many other family members such as grandparents also pay special attention to infant formula. Additionally, some people who plan to have babies in the future will pay special attention to information related to infant formula, even if they have no plans to have babies in the short term or no one close to them is raising children. Therefore, this study did not take the parenting situation as a restriction but rather the respondents’ subjective attention to infant formula. Thirdly, in order to avoid deviations caused by the relevant interests of the respondents, respondents engaged in work related to infant formula and other infant foods and commodities were excluded from the study.

### 3.3. Group Selection

In this study, respondents were classified based on actual risk experience in order to investigate the impact of actual risk experience on anxiety. The classification was based on whether the respondents were victims of or involved in problematic infant formula incidents in reality. Generally, there were three classifications: (1) the babies of the respondents or someone close were the direct victims of problematic infant formula; (2) the babies of the respondents or someone close had not been affected by problematic infant formula, but they were involved in the infant formula incidents (e.g., babies of the respondents or someone close were fed infant formula at the time of the infant formula safety incidents); (3) the babies of the respondents or someone close were neither the direct victims of nor involved in the problematic infant formula incidents. Compared with the third classification, the respondents in the first two classifications were influenced by problematic infant formula. Therefore, actual risk experience in this study was divided into two groups. The group affected by problematic infant formula was the group with actual risk experience, and the group not affected by problematic infant formula was the group without actual risk experience.

### 3.4. Measurements

An online questionnaire was used to gather data in this study. The questionnaire was categorized into three sections (see the Supplementary Materials for details). The first section was a survey of the experience of infant formula safety incidents, aiming to investigate the media risk experience and actual risk experience of the respondents in the three infant formula safety incidents. The second section was the measurement of three variables: anxiety (AN), risk perception (RP), and perceived knowledge gap (PKG), which were measured by Likert’s 7-item scale, with specific definitions. The measurement items and sources are shown in Table 1. The third part was related to demographic variables, mainly related to the gender, age, education level, marriage, and childbearing of respondents.

Based on the literature review, this study defined actual risk experience (ARE) as an individual’s experience of their children (or the children of close people) falling victim to or being involved in problematic infant formula incidents in reality. Based on the group selection above, the first question was designed to examine the respondents regarding their actual risk event experience. Specifically, the three previously mentioned infant formula safety incidents in 2003, 2008, and 2018 were used as materials to investigate the risk experience of the respondents. The item structure of the three events was similar. The options for the item were “Your baby or babies of someone close to you were victims of the problematic infant formula”, and “Your baby or babies of someone close to you had been involved in the problematic infant formula (Your baby or babies of someone close to you were fed infant formula at the time of the infant formula safety incidents)”. These items were designed to distinguish whether the respondent had actual experience of risky events or not.

Media risk experience (MRE) refers to the intensity of the public’s access to risk information from various media [57]. In this study, media risk experience refers to the intensity of the public obtaining information about the risk events of infant formula through various media. The measurement of media risk experience in previous studies was represented by the sum of the intensity of exposure to risk information in various media [58]. This measurement was employed in this study. With reference to the opinions of three experts in related fields, the main types of media exposure of the public during the three infant formula safety incidents were determined, and detailed information on the types of media exposure is presented in the Supplementary Materials. A 7-item Likert scale was employed for the measurement. However, considering that this study involved three incidents, if the sum of the intensity of the three risk experiences is used as the standard, it will lead to weaker results for respondents who have only one or two media risk experiences. To solve this potential problem, the mean value of the respondents’ exposure to various media information intensities in each infant formula safety incident was calculated. If there was no media exposure in one incident, it was recorded as 0 and eliminated in the subsequent calculation. Then, the mean value of the media exposure intensity of the remaining incidents was calculated. The result is the media risk experience.

The questionnaire was formed with the above questions as the main body, and it took 10 to 20 min to answer all the questions. It was written in Chinese, the respondents’ mother tongue, to ensure clear understanding. Since the questionnaire involved the privacy of the respondents, all respondents were required to confirm their informed consent before conducting the survey. Respondents could voluntarily choose whether to leave personal contact information at the end of the questionnaire in order to participate in later research surveys or return visits.

### 3.5. Data Collection

According to Hair [63], when the statistical efficiency is usually assumed to be 80%, considering the model complexity, the minimum sample size required for an R2 value of 0.10 at a significance level of 5% is 90, for PLS-SEM. For this study, this means that at least 90 respondents’ feedback needed to be obtained for each group. Considering the research subjects of this study, we hoped to collect as many samples as possible under the premise of meeting the minimum sample size required for statistical analysis. Therefore, convenient sampling and snowball sampling methods were selected to collect data.

After the preliminary formulation of the questionnaire, the data of 46 respondents were collected through convenient sampling to test the reliability of the questionnaire. Based on the opinions of the respondents, the wording of ambiguous items was modified, and the order of the questionnaire items was adjusted to form a formal questionnaire. The questionnaire was distributed in mainland China via two questionnaire platforms, namely, Wenjuanxing [64] and Tencent Questionnaire [65], which are the two most widely used questionnaire platforms in China. Snowball sampling was used in the distribution of formal questionnaires. At the beginning of the questionnaire, there were questions asking whether the respondents had certain knowledge about infant formula or whether they were interested in it. Only when the respondents confirmed the above questions were follow-up questions asked, by which the respondents were screened.

First, 30 respondents were contacted who met the inclusion criteria through the widely used mother–infant forums Baby Tree [66] and Mamabang [67], as well as mother–infant social media groups on the WeChat and QQ platforms. These respondents were asked to fill in the questionnaire and forward the questionnaire to qualified respondents and social media groups as far as possible. At the same time, the questionnaire was also distributed to the sample database of the questionnaire platform, in the same way, to increase the number of respondents as much as possible. The questionnaire stated that it was hoped that each respondent could forward the questionnaire to individuals or groups who also met the research requirements. The questionnaire was distributed on 1 March 2020. There were no new respondents’ data on 24 March, and there were no new respondents for three consecutive days. The data collection was stopped on 26 March. A total of 506 questionnaires were finally received. Since the online questionnaire quality control and verification function was performed, all of the responses were valid questionnaires. The details of the respondents are shown in Table 2.

### 3.6. Data Analysis

SmartPLS 3.2.2 was employed for data analysis [68]. SmartPLS is a structural equation model statistical analysis software based on the PLS algorithm. The first reason for using PLS-SEM is that, compared with covariance-based structural equation modeling (CBM-SEM), PLS-SEM is more suitable for the analysis of small sample data and does not require the data to be normally distributed. The numbers of respondents in the group without actual risk experience and the group with actual risk experience were 273 and 233, respectively, which met the PLS-SEM requirements for sample size [63]. Moreover, SmartPLS 3.2.2 supports multi-group analysis (MGA) based on non-parametric tests, which facilitates the comparison of models between different groups. Furthermore, media risk experience was one single-item variable in the model, and PLS-SEM is more suitable for processing such data [63]. Data analysis was divided into the following steps:

#### 3.6.1. Common Method Bias Detection

The research data were collected by self-report questionnaires. However, this data collection method may have potential common method bias (CMB) [69]. As ignoring CMB will threaten its validity, this study assessed CMB by calculating the full collinearity before data analysis [70]. The threshold for the full collinearity test was 3.3. If the coefficient value was not greater than 3.3, then the measurement model was free from the concern of CMB.

#### 3.6.2. Measurement Model Testing

For the reflective measurement model, it was necessary to test the reliability and validity. Meanwhile, for single-item indicators, their reliability and validity assessments are not of substantial significance, meaning they are not evaluated. Therefore, this study assessed the reliability and validity of three constructs, namely, perceived knowledge gap (PKG), risk perception (RP), and anxiety (AN).

Generally, an outer loading above the threshold of 0.45 is used to verify the reliability [71]. Cronbach’s α and composite reliability (CR) are commonly used as the criteria for internal consistency assessment. Cronbach’s α assumes that all index variables have the same reliability, and the reliability is estimated based on the correlation between observed indicators. Cronbach’s α values between 0.70 and 0.80 are considered acceptable, and a value greater than 0.80 is excellent. CR takes into account the difference between the outer loadings of index variables, which is comprehensively used to assess the consistency reliability of the measurement model. According to the recommendation of Hair et al., the CR value should generally be higher than 0.70 [72].

Validity was assessed from two aspects: convergent validity and discriminant validity. Convergent validity refers to the similarity of the results when different measurement methods are used to measure the same dimension. Generally, the average variance extracted (AVE) is used as the reference standard. The value of AVE must not be less than 0.5, indicating that the construct can explain more than half of the variance of the index variables [73].

Discriminant validity indicates the degree of difference between the measurement methods of different constructs. Nils and Frederik showed that convergent validity evaluates whether a specific indicator measures its corresponding construct, while discriminant validity assesses whether the indicator inadvertently measures other constructs [74]. The heterotrait–monotrait (HTMT) ratio was used to examine discriminant validity, and a bootstrapping procedure with 5000 resamples was run to obtain the HTMT value. The HTMT confidence interval should not include 1, which suggests that the constructs are empirically distinct, with acceptable discriminant validity.

#### 3.6.3. Measurement Model Invariance Testing

Measurement invariance is used to ensure the validity of outcomes of multi-group comparisons. It guarantees the group differences do not stem from the content and/or meanings of the latent variables, but from the true differences in the structural relations. Researchers suggest that the measurement invariance of composite models (MICOM) procedure in SmartPLS 3.3.2 can be employed to assess the measurement invariance [63].

MICOM is a three-step approach including assessments of configural invariance, compositional invariance, and equal means and variances. When a model using MICOM passes configural invariance (Step 1) and compositional invariance (Step 2), partial measurement invariance is confirmed, which is the precondition for the measure of equal means and variances (Step 3). When partial measurement invariance is established and equal means and variances are obtained, full measurement invariance is established.

#### 3.6.4. Structural Model Testing

Generally, a systematic approach to the assessment of structural models requires establishing collinearity issues, the significance and relevance of the structural model relationships, the level of R^2^, the f^2^ effect size, the predictive relevance Q^2^, and the standardized root mean square residual (SRMR). Variance inflation factor (VIF) criteria were used to examine the potential collinearity within the structural model, and the maximum value needed to be less than 5. To test the hypothesized relationships among the constructs, bootstrapping (5000 subsamples) was performed to assess the significance of path coefficients. R^2^ denotes the predictive accuracy of the model, based on the amount of variation in the dependent variables which will be explained by predictors.

Chin suggested power analysis, where R^2^ values higher than 0.67 are considered as strong coefficients of determination, 0.33 as moderate, and 0.19 as weak [75]. The f^2^ value represents the predictive effects between constructs. According to Chin, f^2^ values of 0.02, 0.15, and 0.35 are regarded as small, medium, and large effects, respectively [75]. Q^2^ is an indicator of predictive relevance, which is obtained by the blindfolding procedure. A Q^2^ larger than 0 indicates the path model has predictive relevance for a reflective construct. SRMR is a model fit measure, whose value is lower than 0.08, indicating that the model fits well.

#### 3.6.5. Mediating Effect Testing

This study followed Zhao’s method [76] to test the mediation effect to test whether risk perception mediates the relationship between perceived knowledge gap and anxiety. First, we evaluated whether the indirect effect was significant. If it was significant, we proceeded to evaluate whether the direct effect is significant. If the direct effect was not significant, it was regarded as a full mediation effect, which means the mediator variable fully explains the relationship between two variables. When the direct effect was significant, it was necessary to assess whether the product of the direct effect and the indirect effect is positive. If it was positive, it was regarded as a complementary mediation effect, while if it was negative, it was regarded as a competitive mediation effect. If it was not a full mediation effect, the method proposed by Hair, J.F et al. was selected for testing variance accounted for (VAF) criteria to better describe the mediation effect [63]. The indirect effect and direct effect were calculated through the formula VAF = indirect effect ÷ (indirect effect + direct effect) to obtain the VAF criteria, that is, the proportion of the indirect effect in the total effect.

#### 3.6.6. Multi-Group Analysis

Based on MICOM analysis, if the result was partial measurement equivalence and complete measurement equivalence, MGA could be performed to compare whether there are significant differences in the paths of the two models.

#### 3.6.7. Moderating Effect Testing

SmartPLS 3.2. 2. can directly deal with the moderating effect of continuous variables. There are three approaches to choose from: (1) the product indicator approach, (2) the orthogonalizing approach, and (3) the two-stage approach. In this study, the variable MRE with a moderating effect needed to be tested as a single-item indicator, and thus the default two-stage approach was selected. If the test results showed that there is a moderating effect, a simple slope analysis was needed to further determine how the moderating variable works.

## 4. Results

### 4.1. Common Method Bias

The results of the full collinearity test for each construct in this study were 1.33, 1.80, and 1.84, which are less than 3.3, indicating the absence of CMB.

### 4.2. Measurement Models

It can be seen from Table 3 that the Cronbach α and CR values in this study were greater than 0.70, most of which exceeded 0.80. With these results, it can be concluded that these constructs had good internal consistency. Moreover, the AVE values of all constructs in the model were above 0.5, indicating good convergence validity. Details of the results of the group with actual risk experience (with ARE) and the group without actual risk experience (without ARE) are shown in Table 3. Table 4 shows the results of the heterotrait–monotrait (HTMT) ratio; the results suggest that the HTMT confidence interval did not contain 1, which indicates that the constructs are empirically distinct with acceptable discriminant validity.

### 4.3. Measurement Model Invariance

Table 5 shows the results of the MICOM assessment, suggesting that anxiety and perceived knowledge gap established full measurement invariance, while risk perception and media risk experience established partial measurement invariance.

### 4.4. Structural Models

**Figure 1:**
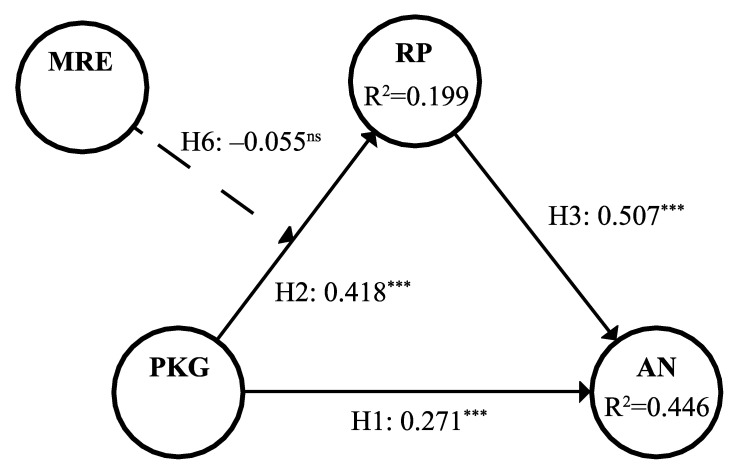
Results of the structural model analysis for the with ARE group. Notes: *** <0.001, ns = not significant.

**Figure 2:**
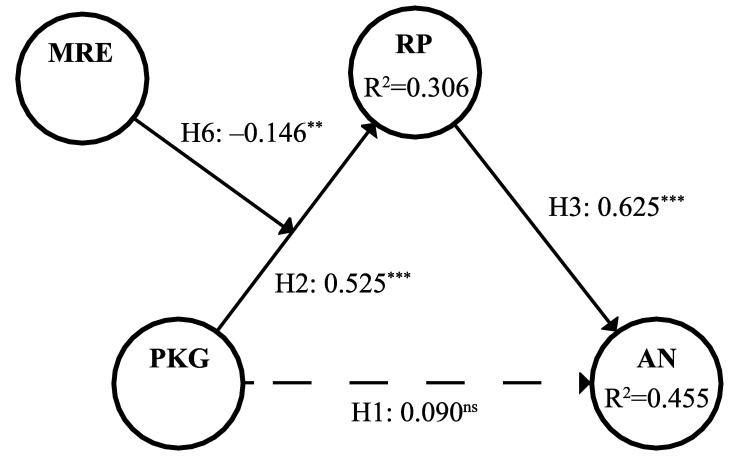
Results of the structural model analysis for the without ARE group. Notes: ** <0.01, *** <0.001, ns = not significant.

Since the results of MICOM suggest the establishment of partial measurement invariance, which does not support the pooled data analysis, we proceeded to assess the structural models of different groups independently. In terms of the collinearity test, the highest value of the construct’s VIF was 1.341, which is less than the standard value of 5, suggesting all the constructs are absent from concerns of collinearity issues. The path coefficient and significance are presented in Table 6, and Figure 1 and Figure 2. It can be seen that in the group with actual experience, all the hypotheses of the paths were valid except the moderating effect of media risk experience, while in the group without actual experience, perceived knowledge gap had no significant impact on anxiety, but all the other hypotheses of the other paths were confirmed. Therefore, hypotheses H2, H3, and H4 were verified, and H1 and H6 were partially valid. The results in Table 6 show that our model moderately explains the variations for both groups with and without actual experience (R^2^_With ARE_ = 0.446, R^2^_Without ARE_ = 0.455). Perceived knowledge gap in the group with actual experience had nearly medium predictive effects on anxiety (f^2^_With ARE_ = 0.109); however, in the group without actual experience, perceived knowledge gap had almost no predictive effects on anxiety (f^2^_Without ARE_ = 0.011). Additionally, the risk perception of both groups had large predictive effects on anxiety (f^2^_With ARE_ = 0.383, f^2^_Without ARE_ = 0.535). As can be seen, the Q^2^ values of all constructs were above 0, which is indicative of a predicative relevance. The estimated SRMR values of both groups were 0.079, lower than the threshold of 0.08, indicating that our model has a good fit.

### 4.5. Mediating Effects

According to the results in Table 6, the group without actual experience had a full mediation effect, and the group with actual experience had a complementary mediation effect. To better describe the mediation effect of the group with actual experience, the VAF was calculated. The results show that the VAF of the actual experience group was 43.89%, suggesting that hypothesis H5 was supported.

### 4.6. Multi-Group Analysis

The results for the multi-group analysis are shown in Table 7. There were significant differences between the two groups in the effect of perceived knowledge gap on anxiety. This effect in the group with actual experience was significantly higher than in the group without actual experience, and there was no significant difference between the other paths.

Regarding the differences in the variables of the two groups, according to the results of MICOM step 3 in Table 5, there was no significant difference between anxiety and perceived knowledge gap between the two groups. Meanwhile, for media risk experience, risk perception in the group without actual experience was significantly lower than in the group with actual experience.

### 4.7. Moderating Effects

**Figure 3:**
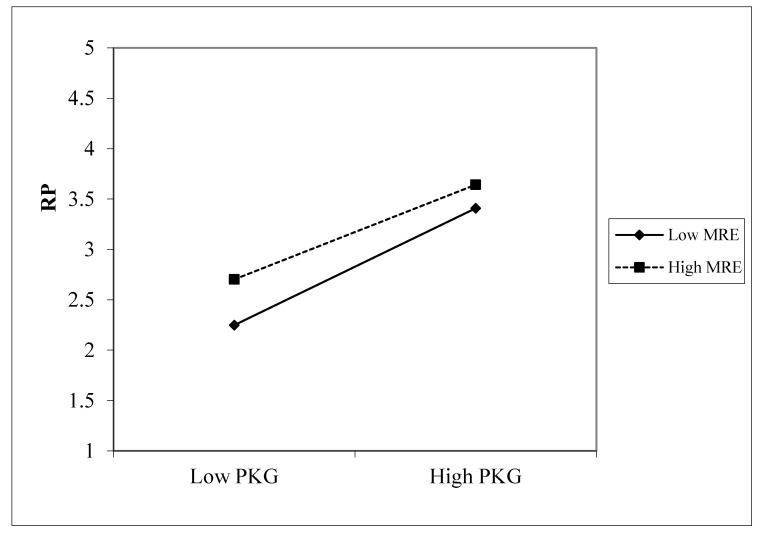
Moderation effect.

According to the results in Table 6, the effect of the interaction between media risk experience and perceived knowledge gap on anxiety was not significant in the group with actual experience, but it was significant in the group without actual experience, indicating that media risk experience played a moderating role in the group without actual experience. Therefore, it was necessary to further analyze the moderating effect of media risk experience in the group without actual experience. According to Gardner et al., a weakening moderating relationship is regarded as the difference in the coefficient directionality and the moderating relationship between independent variables [77]. As can be seen from the simple slope analysis in Figure 3, media risk experience had a weakening moderating relationship between perceived knowledge gap and risk perception. In other words, when media risk experience was lower, perceived knowledge gap was more likely to stimulate risk perception, but this effect did not play a role in the group with actual experience.

## 5. Discussion

Based on the above research results, it was found that in terms of the safety issue of infant formula, perceived knowledge gap had an indirect impact on anxiety through the mediating effect of risk perception. This result partially verifies the basic model established in this study. However, through the comparison of groups with and without actual risk experience, and the test of the moderating effect of media experience, it was found that actual risk experience and media risk experience played an important role. For the group without actual risk experience, the relationship between perceived knowledge gap and anxiety was completely mediated by risk perception; that is, perceived knowledge gap had no direct effect on anxiety, while media risk experience mediated the impact of perceived knowledge gap on risk perception. The lower the media risk experience, the stronger the impact of perceived knowledge gap on risk perception. For the group with actual risk experience, perceived knowledge gap had a positive impact on anxiety, and risk perception had a complementary mediation effect, while the moderating effect of media risk experience was not significant.

With regard to the effect of perceived knowledge gap on risk perception, the research findings indicate that it is universal to generate a risk perception mechanism when individuals perceive a lack of knowledge of risk events, which is in line with the findings of Shakeri et al. and Liao et al. [37,38]. In the face of risk events, individuals will judge the degree of risk according to their knowledge base and then generate a psychological response and behavioral decision making accordingly, which is an analytical process. Moreover, the impact of risk perception on anxiety is consistent with the RISP model proposed by Griffin, indicating that individuals will have negative emotions such as anxiety after perceiving risk. Perceived knowledge gap affected anxiety through risk perception, which was valid in both groups, indicating that this structure has a certain degree of stability and is not affected by risk experience. However, the results of this study are inconsistent with a previous study in terms of the impact of perceived knowledge gap on anxiety [27], because a significant effect was only detected in the group with actual risk experience, indicating that an experienced food risk in the past will persist. When a new food safety incident of the same type occurs, perceived knowledge gap has a direct impact on anxiety, which is complementary to the mediating role of anxiety. This path of influence is probably heuristic; thus, the moderating effect of media risk experience does not play a significant role, which suggests that the role of actual risk experience replaces the role of media risk experience. Individuals who have experienced actual food safety risks are more inclined to refer to their own experience, and they will ignore the media experience to a certain extent, leading to a weakening of the influence of media experience. This reveals that actual risk experience takes precedence over media risk experience in the individual’s emotional response. Overall, these results show that the views of Knuth et al. are reasonable [46]. For most people who have not experienced food safety risks, perceived knowledge gap will not directly lead to negative emotions such as anxiety. Instead, individuals will judge the distance between the risk and themselves according to the previous experience gained from media in order to perceive and judge the risk. It is plausible that this process will elicit negative emotions. Therefore, risk perception plays a complete mediating role in most cases. For people who have had actual risk experience, actual risk experience will weaken or even replace the mediating role of media experience, enabling individuals to establish an empirical and heuristic relationship between perceived knowledge gap and anxiety, and to generate emotional feedback more directly. This result indicates that an actual food safety risk experience should be distinguished from the food safety risk experience obtained from the media since the two have different effects on individuals facing food safety risks.

Additionally, according to the comparison results of the mean values of various factors in the MGA, it was found that there was no significant difference in perceived knowledge gap and anxiety between the two groups, but the group without actual risk experience was significantly lower than the group with actual risk experience in media risk experience and risk perception. This result suggests that individuals with actual risk experience may pay more attention to information in the media in previous risk events, meaning they have stronger media risk experience. However, they do not reduce the level of their perceived knowledge gap, even though they accumulate more risk-related knowledge. This indicates that the strength of risk experience and the level of knowledge cannot determine the strength of the perceived knowledge gap. Perceived knowledge gap is an individual’s subjective judgment on their risk-related knowledge. Therefore, it is this subjective attitude that affects risk perception and anxiety, not the level of knowledge itself. The difference in risk perception revealed that actual risk experience will make individuals more sensitive to risk perception, and it is easy to stimulate a higher level of risk perception when facing food safety risks.

Based on the above analysis, it is clear that in food safety risk incidents, such as infant formula safety incidents, reducing the public’s perceived knowledge gap is essential to relieve anxiety. In other words, it is quite necessary to popularize more knowledge among the public. However, perceived knowledge gap is the lack of knowledge perceived by the public rather than the knowledge level itself. Therefore, it is particularly important to reduce the level of perceived knowledge gap by analyzing the correlation between new food safety risk events and previous events through media reports.

Meanwhile, media reports should be timely and detailed to reduce the public’s risk perception and decrease public anxiety further. For groups without actual risk experience, timely and detailed reports can also enhance the effect of media risk experience and reduce the impact of perceived knowledge gap on risk perception through the moderating effect of media risk experience.

This study has some limitations, as expected, and despite the comparison of different groups with actual risk experience and without actual risk experience, it was impossible to examine whether the group with actual risk experience responded more quickly to anxious situations in this cross-sectional study. This is very important for the relevant research on the generation mechanism of social emotions in food safety risk events. Secondly, this study classified the respondents according to whether they had actual risk experience. However, there may be other differences due to the severity of the impact, such as the psychological distance of the risk. Therefore, a more detailed classification should be considered in future research.

## 6. Conclusions

The effects of perceived knowledge gap, risk perception, media risk experience, and actual risk experience on public anxiety in infant formula safety incidents were explored in this study. The results obtained from the data analysis of 506 questionnaires show that for the group without actual risk experience, perceived knowledge gap positively influenced risk perception, and risk perception had positive effects on anxiety, while risk perception played a complete mediating role, and media risk experience played a weakening moderating role in the impact of perceived knowledge gap on risk perception. For the group with actual risk experience, risk perception had a complementary mediation effect in the effect of perceived knowledge gap on anxiety, and the moderating effect of media risk experience was insignificant. This suggests that the overall perceived knowledge gap affects anxiety through the mediation effect of risk perception. However, the actual risk experience will bridge the relationship between perceived knowledge gap and anxiety, and this path may be heuristic and will replace the role of media risk experience.

We encourage future research efforts to channel into three directions. First of all, the model in this study was established based on infant formula safety incidents; however, the generalization and application scope of the model should take more contexts into consideration and be validated in future study. Second, in view of the first research limitation mentioned above, it could be worthwhile to investigate whether there is a difference in reaction speed between groups with and without actual risk experience in food safety incidents, and whether there is a difference in the cycle of social emotions through longitudinal research. Lastly, as mentioned earlier, reducing the intensity and influence of anxiety is inseparable from the support of media reports. However, the form of reporting (such as different narrative frameworks and different information release strategies) can effectively reduce the impact of perceived knowledge gap, risk perception, and media risk experience on anxiety, which is worthy of further research and discussion.

## Figures and Tables

**Table 1:** Definition and measurement of variables.

Constructs	Definition	Question	Source
Anxiety	Anxiety is defined as nervousness, apprehension, worry, and fear about the occurrence of milk powder accidents and the subsequent serious consequences (Hmielowski JD et al., 2018) [7]	AN1: I am always afraid of potential risks in infant formulas on the market.	Lagoe et al. [59] Lucock and Morley [60]
		AN2: When I heard about the infant formula safety incident, I would worry that the infants and young children in the family or myself would be at risk.	
		AN3: It scares me to think that the infants and young children in my family or myself may be involved in the fake infant formula.	
		AN4: I often worry that the infants in my family or myself are threatened by the fake infant formula.	
Perceived knowledge gap	Perceived knowledge gap is defined as the degree to which the public perceives their knowledge to be insufficient in the context of infant formula risk events (Shakeri S et al., 2018) [38]	PKG1: I don’t think I have enough knowledge about the safety of infant formula.	Erci [61] Shakeri et al. [38]
		PKG2: I am not clear about the relevant information on infant formula.	
		PKG3: I still have many questions about the safety issue of the current infant formula.	
		PKG4: I still don’t know enough about the risks of infant formula.	
Risk perception	Risk perception is defined as the public’s subjective judgment on the likelihood of an infant formula safety incident and the severity of its consequences (Griffin et al., 2008) [16]	RP1: I am worried that the infants and young children in the family or myself will suffer from the fake infant formula.	Kellens W et al. [62] Wu and Li [58]
		RP2: I think the fake infant formula will have fatal consequences for infants and young children.	
		RP3: When there is an infant formula safety incident, I cannot ensure that I can get enough warning and protection.	
		RP4: The safety of infant formula is a potential threat to the healthy and orderly development of society.	

**Table 2:** Descriptive statistics of respondents’ demographics.

Variables		*n*	%
Gender	Male	98	19.400
	Female	408	80.600
Age	18–25	79	15.600
	26–35	182	36.000
	36–45	175	34.600
	46–55	67	13.200
	56–65	3	0.600
Education Level	High School or Less	89	17.589
	Junior College	110	21.739
	Bachelor Degree	226	44.664
	Master Degree	81	16.008
Disposable Income Per Month	3000 yuan or Below	134	26.482
	3000 yuan–5000 yuan	189	37.352
	5000 yuan–10,000 yuan	125	24.704
	10,000 yuan and Above	58	11.462
Parenting Situation	Childless	142	28.100
	Child-Rearing	364	71.900
Risk Experience	with Actual Risk Experience	233	46.050
	without Actual Risk Experience	273	53.950

**Table 3:** Results for the measurement models.

Constructs	Loadings		CR		Cronbach’s α		AVE	
	with ARE	without ARE	with ARE	without ARE	with ARE	without ARE	with ARE	without ARE
Anxiety			0.899	0.905	0.849	0.859	0.691	0.705
AN1	0.744	0.755						
AN2	0.850	0.872						
AN3	0.834	0.873						
AN4	0.891	0.852						
Perceived knowledge gap			0.885	0.909	0.873	0.868	0.660	0.714
PKG1	0.754	0.803						
PKG2	0.793	0.836						
PKG3	0.845	0.881						
PKG	0.853	0.857						
Risk perception			0.819	0.874	0.711	0.807	0.532	0.634
RP1	0.772	0.743						
RP2	0.616	0.802						
RP3	0.776	0.794						
RP4	0.741	0.843						

**Table 4:** Results of HTMT ratio.

Relationships	Confidence Interval (95%)	
	with ARE	without ARE
PKG–AN	[0.414, 0.659]	[0.298, 0.585]
RP–AN	[0.645, 0.900]	[0.711, 0.876]
RP–PKG	[0.355, 0.646]	[0.470, 0.691]

**Table 5:** Results of invariance measurement testing (with ARE–without ARE).

Constructs	Configural Invariance	Compositional Invariance		Partial Measurement Invariance	Equal Mean Assessment			Equal Variance Assessment			Full Measurement Invariance
		Original Correlation	Confidence Interval		Difference	Confidence Interval	Equal	Difference	Confidence Interval	Equal	
AN	Yes	1.000	[0.998, 1.000]	Yes	0.103	[−0.171, 0.175]	Yes	0.046	[−0.273, 0.0.267]	Yes	Yes
MRE	Yes	1.000	[1.000, 1.000]	Yes	0.385	[−0.180, 0.177]	No	−0.128	[−0.177, 0.173]	Yes	No
PKG	Yes	1.000	[0.997, 1.000]	Yes	−0.003	[−0.172, 0.175]	Yes	−0.063	[−0.261, 0.258]	Yes	Yes
RP	Yes	0.997	[0.996, 1.000]	Yes	0.254	[−0.169, 0.174]	No	−0.410	[−0.310, 0.300]	No	No

**Table 6:** Results for structural models.

Hypothesis	Relationships	Path Coefficient		T Statistics		Supported		R^2^		f^2^		Q^2^	
		with ARE	without ARE	with ARE	without ARE	with ARE	without ARE	with ARE	without ARE	with ARE	without ARE	with ARE	without ARE
H1	PKG ≥ AN	0.271 ***	0.090 ^ns^	4.187	1.482	Yes	No	0.446	0.455	0.109	0.011	0.296	0.309
H3	RP ≥ AN	0.507 ***	0.625 ***	8.066	12.789	Yes	Yes			0.383	0.535		
H4	PKG ≥ RP ≥ AN	0.212 ***	0.328 ***	4.897	8.159	Yes	Yes			-	-		
H2	PKG ≥ RP	0.418 ***	0.525 ***	7.568	11.505	Yes	Yes	0.199	0.306	0.217	0.393	0.091	0.178
H6	PKG × MRE ≥ RP	0.055 ^ns^	−0.146 **	0.792	3.092	No	Yes			0.005	0.038		
-	MRE ≥ RP	0.143 **	0.172 ***	2.197	3.550	-	-			0.025	0.042		

Notes: ** *p* < 0.01, *** *p* < 0.001, ns = not significant (*p* > 0.05).

**Table 7:** Results of MGA.

Hypothesis	Relationships	Path Coefficient Difference (with ARE–without ARE)	PLS-MGA *p* Values	Permutation *p* Values	Supported
H5	PKG ≥ AN	0.181	0.048	0.049	Yes
-	PKG ≥ RP	−0.107	0.130	0.140	-
-	RP ≥ AN	−0.118	0.143	0.148	-
-	MRE ≥ RP	−0.030	0.716	0.699	-
-	PKG × MRE ≥ RP	0.090	0.283	0.285	-

Notes: Significance level is 0.05.

## Data Availability

The data presented in this study can be provided upon request to the corresponding author. For ethical reasons, these data cannot be made public.

## Supplementary Materials

The following are available online at https://www.mdpi.com/article/10.3390/healthcare10010138/s1, Main items of the questionnaire.

## Abbreviations

PKG	Perceived knowledge gap
RP	Risk perception
AN	Anxiety
MRE	Media risk experience
ARE	Actual risk experience
