# Supplementary material for: The Effect of Experience on Anxiety in Food Safety Incidents—An Empirical Study on Infant Formula Safety Incidents in China
Source: Healthcare (Basel). 2022 Jan 12;10(1):138. doi: 10.3390/healthcare10010138 (PMC8776098; doi:10.3390/healthcare10010138)
Supplement: Supplementary file 1 [file healthcare-10-00138-s001.zip › healthcare-1500406-Supplementary.pdf]

Supplementary material : Main items of the questionnaire

Part I

Incident 1: From 2003 to 2004, a large number of fake infant formula flowed into rural sales points in Fuyang, Anhui Province. "Toxic milk powder" led to the growth stagnation of infants and the decline of immunity. 189 infants in Fuyang suffered from mild to moderate malnutrition and 12 infants died.

1.1 When the above Incident 1 occurred, which of the following statements best describes your situation?

☐ Your baby or babies of someone close to you were victims of the infant formula safety incident.

☐ Your baby or babies of someone close to you had been involved in the problematic infant formula (Your baby or babies of someone close to you were fed infant formula at the time of the infant formula safety incidents).

☐ None of the above.

1.2 During the above Incident 1, how much did you know about the incident through various media? (A 7-point Likert scale ranging from 1 "not at all" to 7 "very well")

☐<br>1	☐<br>2	☐<br>3	☐<br>4	☐<br>5	☐<br>6	☐<br>7

1.3 How often do you use the following media to access information about reports in Incident 1? (A 7-point Likert scale ranging from 1 "never" to 7 "very frequently")

Television	☐<br>1	☐<br>2	☐<br>3	☐<br>4	☐<br>5	☐<br>6	☐<br>7
Radio broadcast	☐<br>1	☐<br>2	☐<br>3	☐<br>4	☐<br>5	☐<br>6	☐<br>7
Newspaper and magazines	☐<br>1	☐<br>2	☐<br>3	☐<br>4	☐<br>5	☐<br>6	☐<br>7
Web portal	☐<br>1	☐<br>2	☐<br>3	☐<br>4	☐<br>5	☐<br>6	☐<br>7
Online forums	☐<br>1	☐<br>2	☐<br>3	☐<br>4	☐<br>5	☐<br>6	☐<br>7
Instant messaging tools	☐<br>1	☐<br>2	☐<br>3	☐<br>4	☐<br>5	☐<br>6	☐<br>7
Social media	☐<br>1	☐<br>2	☐<br>3	☐<br>4	☐<br>5	☐<br>6	☐<br>7

Incident 2: In 2008, some batches of infant formula produced by the domestic brand Sanlu were detected to contain the chemical raw material melamine. The toxic infant formula caused infants to suffer from kidney stones, renal failure, and other diseases. It was reported that 39000 infants were hospitalized and 4 infants died.

2.1 When the above Incident 2 occurred, which of the following statements best describes your situation?

☐ Your baby or babies of someone close to you were victims of the infant formula safety incident.

☐ Your baby or babies of someone close to you had been involved in the infant formula safety incident (Your baby or babies of someone close to you were fed infant formula at the time of the infant formula safety incidents.)

☐ None of the above.

2.2 During the above Incident 2, how much did you know about the incident through various media? (A 7-point Likert scale ranging from 1 "not at all" to 7 "very well")

☐ 1	☐ 2	☐ 3	☐ 4	☐ 5	☐ 6	☐ 7

2.3 How often do you use the following media to access information about the Incident 2? (A 7-point Likert scale ranging from 1 "never" to 7 "very frequently")

Television	☐ 1	☐ 2	☐ 3	☐ 4	☐ 5	☐ 6	☐ 7
Radio broadcast	☐ 1	☐ 2	☐ 3	☐ 4	☐ 5	☐ 6	☐ 7
Newspaper and magazines	☐ 1	☐ 2	☐ 3	☐ 4	☐ 5	☐ 6	☐ 7
Web portal	☐ 1	☐ 2	☐ 3	☐ 4	☐ 5	☐ 6	☐ 7
Online forums	☐ 1	☐ 2	☐ 3	☐ 4	☐ 5	☐ 6	☐ 7
Instant messaging tools	☐ 1	☐ 2	☐ 3	☐ 4	☐ 5	☐ 6	☐ 7
Social media	☐ 1	☐ 2	☐ 3	☐ 4	☐ 5	☐ 6	☐ 7

Incident 3: From 2015 to 2019, media including the Beijing News, CCTV Finance Channel, and CCTV News Live had repeatedly reported the safety risks of imported overseas infant formula. The repeated incidents of producing and selling fake infant formula have caused serious distress to consumers. In particular, the report that Spain seized fake infant formula that was original to be shipped to China in 2018 caused widespread concern among the

consumers who were willing to pay a premium for imported infant formula.

3.1 When the above Incident 3 occurred, which of the following statements best describes your situation?

- ☐ Your baby or babies of someone close to you were victims of the infant formula safety incident.
- ☐ Your baby or babies of someone close to you had been involved in the infant formula safety incident (Your baby or babies of someone close to you were fed infant formula at the time of the infant formula safety incidents.)
- ☐ None of the above. You have never been affected by the fake infant formula.

3.2 During the above Incident 3, how much did you know about the incident through various media? (A 7-point Likert scale ranging from 1 “not at all” to 7 “very well”)

☐<br>1	☐<br>2	☐<br>3	☐<br>4	☐<br>5	☐<br>6	☐<br>7

3.3 How often do you use the following media to access information about Incident 3? (A 7-point Likert scale ranging from 1 “never” to 7 “very frequently”)

Television	☐<br>1	☐<br>2	☐<br>3	☐<br>4	☐<br>5	☐<br>6	☐<br>7
Radio broadcast	☐<br>1	☐<br>2	☐<br>3	☐<br>4	☐<br>5	☐<br>6	☐<br>7
Newspaper and magazines	☐<br>1	☐<br>2	☐<br>3	☐<br>4	☐<br>5	☐<br>6	☐<br>7
Web portal	☐<br>1	☐<br>2	☐<br>3	☐<br>4	☐<br>5	☐<br>6	☐<br>7
Online forums	☐<br>1	☐<br>2	☐<br>3	☐<br>4	☐<br>5	☐<br>6	☐<br>7
News apps	☐<br>1	☐<br>2	☐<br>3	☐<br>4	☐<br>5	☐<br>6	☐<br>7
Instant messaging tools	☐<br>1	☐<br>2	☐<br>3	☐<br>4	☐<br>5	☐<br>6	☐<br>7
Social media	☐<br>1	☐<br>2	☐<br>3	☐<br>4	☐<br>5	☐<br>6	☐<br>7

Part II

(A seven-point Likert scale was employed, with 1 corresponding to strongly disagree, 7 strongly agree.)

Anxiety

- I am always afraid of potential risks in infant formulas on the market.

☐	☐	☐	☐	☐	☐	☐
1	2	3	4	5	6	7

- When I heard about the infant formula incident, I would worry that my baby or babies of someone close to me would be at risk.

☐	☐	☐	☐	☐	☐	☐
1	2	3	4	5	6	7

- It scares me to think that my baby or babies of someone close to me may be involved in the problematic infant formula.

☐	☐	☐	☐	☐	☐	☐
1	2	3	4	5	6	7

- I often worry that my baby or babies of someone close to me are threatened by the problematic infant formula.

☐	☐	☐	☐	☐	☐	☐
1	2	3	4	5	6	7

Perceived knowledge gap

- I don't think I have enough knowledge about the safety of infant and toddler formulas.

☐	☐	☐	☐	☐	☐	☐
1	2	3	4	5	6	7

- I am not clear about the status quo of infant and toddler formulas.

☐	☐	☐	☐	☐	☐	☐
1	2	3	4	5	6	7

- I still have many questions about the safety issue of the current infant milk formula

☐	☐	☐	☐	☐	☐	☐
1	2	3	4	5	6	7

- I still don’t know enough about the risks of infant and toddler formulas.

☐	☐	☐	☐	☐	☐	☐
1	2	3	4	5	6	7

Risk perception

- I am worried that my baby or babies of someone close to me will suffer from the problematic infant formula.

☐	☐	☐	☐	☐	☐	☐
1	2	3	4	5	6	7

- I think the problematic infant formula will have fatal consequences for infants and young children.

☐	☐	☐	☐	☐	☐	☐
1	2	3	4	5	6	7

- When there is an infant formula incident, I cannot ensure that I can get enough warning and protection.

☐	☐	☐	☐	☐	☐	☐
1	2	3	4	5	6	7

- The safety of infant and toddler formulas is a potential threat to the health and orderly development of society.

☐	☐	☐	☐	☐	☐	☐
1	2	3	4	5	6	7

Part III

- Your gender: ______
- Your age: ______
- Your education level: ______
- Your disposable income per month: _______
- Childbearing: ______
